# Supplementary material for: Hall effect in charged conducting ferroelectric domain walls
Source: Nat Commun. 2016 Dec 12;7:13764. doi: 10.1038/ncomms13764 (PMC5159852; doi:10.1038/ncomms13764)
Supplement: Supplementary Information — Supplementary Figures 1-3 [file ncomms13764-s1.pdf]

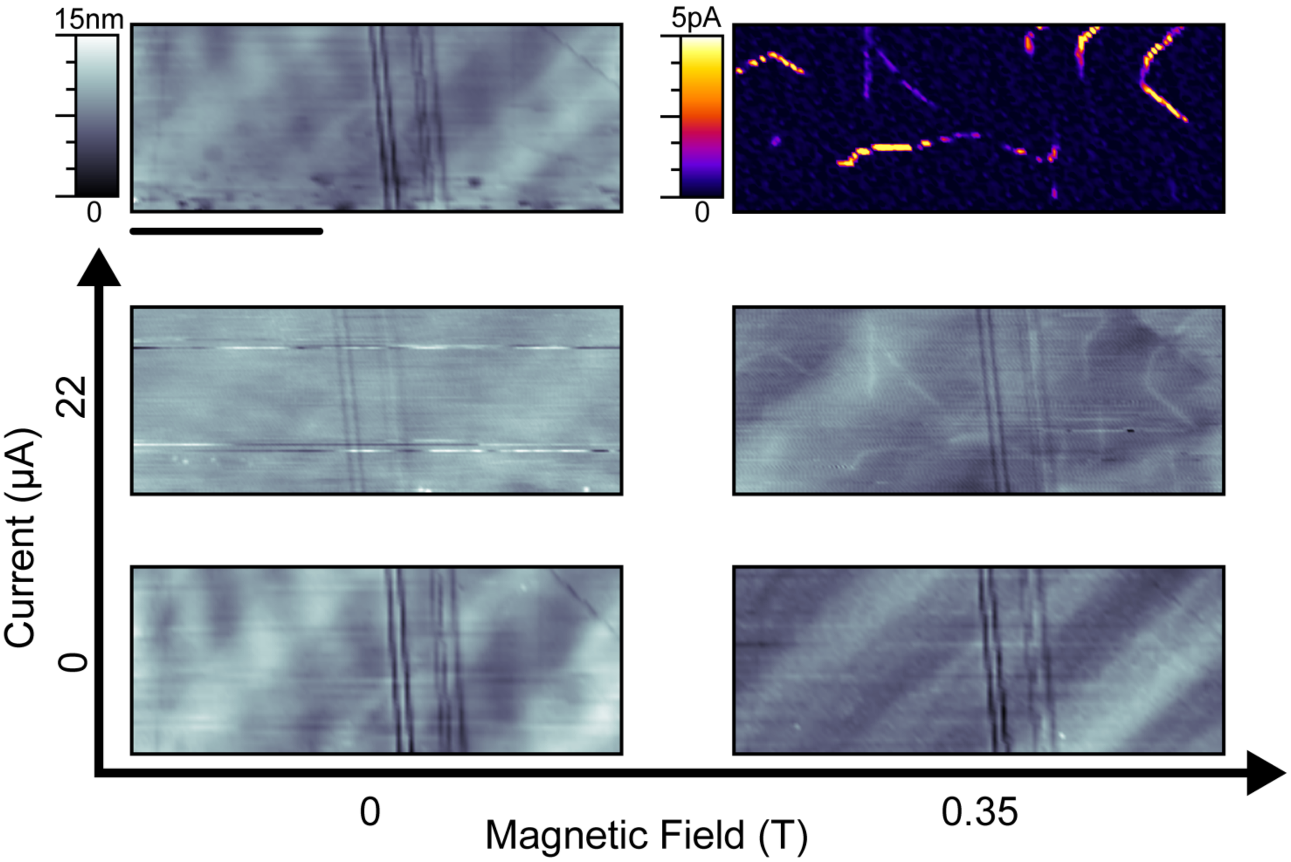

Supplementary Figure 1 | Topography maps acquired during nanoscale Hall Effect imaging at functional domain walls. Data shown in the main text is a derivative map, here we show the unfiltered topography images. Scale bar is 5μm.

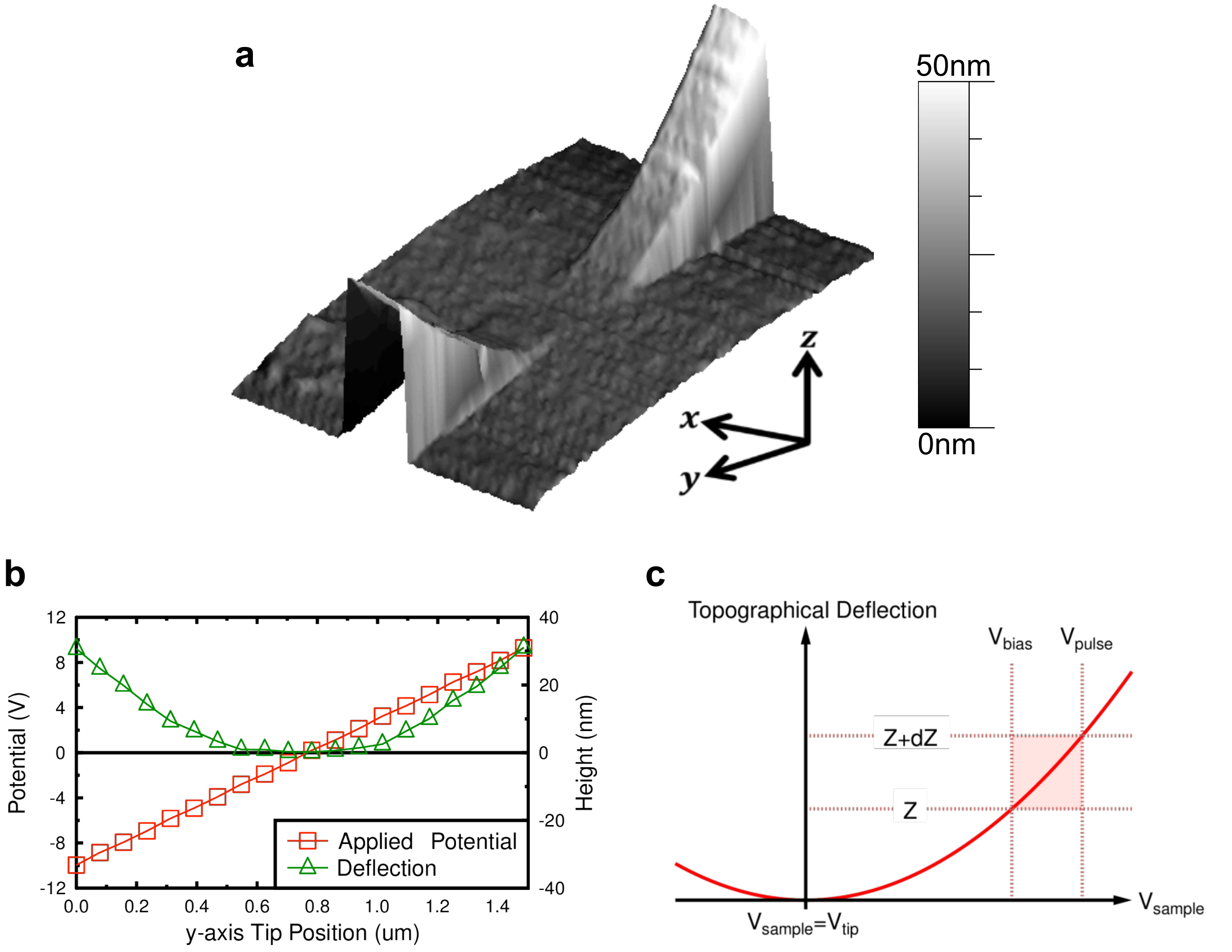

Supplementary Figure 2 | Quadratic behavior of calibration measurements recorded in absence of a dc background. a, A tapping mode atomic force microscopy topography map of a gold-coated $SrTiO_3$ single crystal showing the measured signal as linearly increasing voltage pulses, from -10V to 10V, were applied in absence of a DC background. **b,** The value of the applied voltage pulse plotted as a function of tip position along the scan and the corresponding topographical deflection are plotted on the same axis to convey the correlation between signals. An approximately quadratic topographic signal is observed in response to a linearly varied potential. **c,** Schematic representation of the behavior of the measured topographic deflection as a function of the applied potential, wherein V_{sample} and V_{tip} represent the potential at the sample and of the tip, V_{bias} the background dc potential, V_{pulse} the increase in potential on application of a voltage pulse, Z the measured topographic height and dZ is a increase in measured height due to application of voltage pulse. Within the region where sample and tip potential equate, the response is quadratic such that any increase in the magnitude of the sample potential, regardless of sign, will result in positive increase in the measured topography. Application of a dc background, shifts along the plot to the region represented by the shaded box, where the topographic response is approximately linear.

$$\mathbf{F}_{\mathrm{m}} = q(\mathbf{v} \times \mathbf{B})$$

$$\mathbf{F}_{\mathrm{m}} = +q\left(\begin{pmatrix}\mathbf{v}_{\mathrm{x}}\\ \mathbf{v}_{\mathrm{y}}\\ \mathbf{v}_{\mathrm{z}}\end{pmatrix} \times \begin{pmatrix}\mathbf{B}_{\mathrm{x}}\\ \mathbf{B}_{\mathrm{y}}\\ \mathbf{B}_{\mathrm{z}}\end{pmatrix}\right)$$

$$\mathbf{F}_{\mathrm{m}} = +q\begin{pmatrix}\mathbf{v}_{\mathrm{y}}\mathbf{B}_{\mathrm{z}} - \mathbf{v}_{\mathrm{z}}\mathbf{B}_{\mathrm{y}}\\ \mathbf{v}_{\mathrm{z}}\mathbf{B}_{\mathrm{x}} - \mathbf{v}_{\mathrm{x}}\mathbf{B}_{\mathrm{z}}\\ \mathbf{v}_{\mathrm{x}}\mathbf{B}_{\mathrm{y}} - \mathbf{v}_{\mathrm{y}}\mathbf{B}_{\mathrm{x}}\end{pmatrix}$$

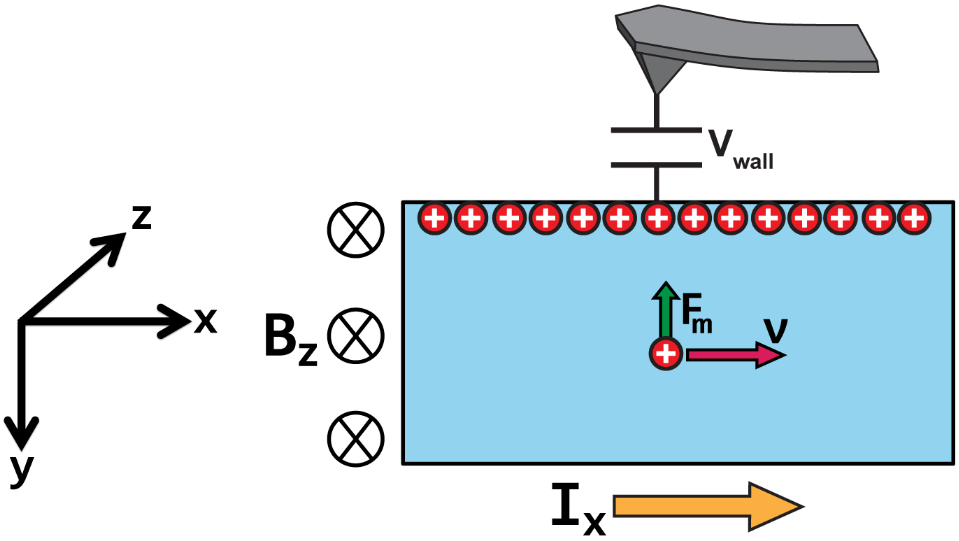

$$\mathbf{F}_{\mathrm{m}} = +q\left(\begin{pmatrix}1\\0\\0\end{pmatrix} \times \begin{pmatrix}0\\0\\1\end{pmatrix}\right)$$

$$\mathbf{F}_{\mathrm{m}} = +q\begin{pmatrix}0\\-1\\0\end{pmatrix}$$

Supplementary Figure 3 | Schematic of the experimental geometry to illustrate direction of Hall Potential at the studied walls. From the sign of the potential on the tip, and the directions of applied current and magnetic field, the carrier type responsible for the observed tip deflection can be determined. The vector relationships for determining the carrier type are shown. The Lorentz force, $\mathbf{F}_{\mathrm{m}}$, is calculated by considering the relative directions of the carrier drift velocity, **v**, the magnetic field, $\mathbf{B}_{\mathrm{z}}$, and the current flow, $\mathbf{I}_{\mathrm{x}}$.
